# Tensor Decomposition for Colour Image Segmentation of Burn Wounds

**DOI:** 10.1038/s41598-019-39782-2

**Published:** 2019-03-01

**Authors:** Marco D. Cirillo, Robin Mirdell, Folke Sjöberg, Tuan D. Pham

**Affiliations:** 1 Department of Biomedical Engineering, Linköping University, Linköping, Sweden; 2 The Burn Centre, Department of Plastic Surgery, Hand Surgery, and Burns, Linköping University, Linköping, Sweden; 3 Department of Clinical and Experimental Medicine, Linköping University, Linköping, Sweden

## Abstract

Research in burns has been a continuing demand over the past few decades, and important advancements are still needed to facilitate more effective patient stabilization and reduce mortality rate. Burn wound assessment, which is an important task for surgical management, largely depends on the accuracy of burn area and burn depth estimates. Automated quantification of these burn parameters plays an essential role for reducing these estimate errors conventionally carried out by clinicians. The task for automated burn area calculation is known as image segmentation. In this paper, a new segmentation method for burn wound images is proposed. The proposed methods utilizes a method of tensor decomposition of colour images, based on which effective texture features can be extracted for classification. Experimental results showed that the proposed method outperforms other methods not only in terms of segmentation accuracy but also computational speed.

## Introduction

Burns are among the most life-threatening of traumatic injuries^1^. Severe burns constitute a major crisis for the public health with an implication to a considerable health-economic impact, as they can cause substantial morbidity and mortality through infection, sepsis, organ failure, and death, where the mortality rate has been reported between 1.4% and 18% (maximum 34%)^2^.

The World Health Organization has guidelines for burn treatment that, at least, there must be one bed in a burn unit for each 500,000 inhabitants^3^. A burn unit covers a wide geographic area and a burnt patient is usually diagnosed by non-specialized burn experts. In Sweden, for example, there are only two burn centres in the whole country: one located in Linköping and the other one in Uppsala.

Burns are categorized into several types by depth: 1^st^ degree, superficial partial-thickness, deep partial-thickness, and full-thickness burns^4^. When calculating the percentage of total body surface area (%TBSA) burnt, only superficial partial-thickness burns and deeper are included in the area calculation, while 1^st^ degree burns (with intact epidermis) are excluded. To provide the right clinical treatment to a burn patient, the %TBSA of the burn must be calculated as it dictates the early fluid resuscitation. The actual %TBSA is also useful for later surgical planning and for estimating mortality using the revised Baux-score, which has proven to be a reliable predictor of both mortality and morbidity even though the %TBSA used is estimated through clinical means^5^. Given limited burn treatment facilities over a large geographic environment, especially in middle and low income countries, and the importance of burn area calculation, the demand for developing automated methods for accurate and objective assessment of burn parameters have been increasingly realized in burn research.

This project proposes a new method for the burn-wound image segmentation using a method of tensor decomposition that can extract effective luminance-colour texture features for classification of burn and non-burn areas. The tensor decomposition is a generalization of PCA. Both PCA and ICA are still actively applied to image segmentation^6,7^. The remaining of this paper is organized as follows. Section 2 reviews works relating to the proposed method. Section 3 describes the materials and models of the proposed method. Section 4 presents the experimental results and discussion. Finally, Section 5 summarizes the research finding and suggests issues for future research.

## Related Works

Segmentation is one of the major research areas in image processing and computer vision. The goal of image segmentation is to extract the region of interest in an image that includes a background and other non-interest objects. There are many different techniques developed to accomplish the segmentation, such as edge detection, histogram thresholding, region growing, active contours or snake algorithm, clustering, and machine-learning based methods, as reviewed in^8,9^, which extract the characteristics that describe image such as: luminance, brightness, colour, texture, and shape^9,10^. The combination of these properties, where applicable, is expected to provide better segmentation results than those that utilize just one or fewer.

Deng and Manjunath^11^ proposed the JSEG method, which separates the segmentation process into two stages: colour quantization and spatial segmentation. In the first stage, colours in the image are quantized to several representative classes that can be used to differentiate regions in the image. This quantization is performed in the colour space without considering the spatial distributions of the colours. The image pixel values are then replaced by their corresponding colour class labels, thus forming a class-map of the image. The class-map can be viewed as a special kind of texture composition. In the second stage, spatial segmentation is performed directly on this class-map without considering the corresponding pixel colour similarity. Cucchiara *et al*.^12^ developed a segmentation method for extracting skin lesions based on a recursive version of the fuzzy *c*-means algorithm^13^ (FCM) for 2D colour histograms constructed by the principal component analysis (PCA) of the CIELab colour space.

Acha *et al*.^14^ worked with the CIELuv colour space for the image segmentation by extracting the colour-texture information from a 5 × 5 pixel area around a point that the user selects with the mouse. These features are combined, and the Euclidean distance between the previously chosen area and the others is calculated to classify two regions of burn and non-burn, using the Otsu’s thresholding method^15^. Gomez *et al*.^16^ developed an algorithm based on the CIELab colour space and independent histogram pursuit (IHP) to segment skin lesions images. The IHP is composed by 2 steps: firstly, the algorithm finds a combination of spectral bands that enhance the contrast between healthy skin and lesion; secondly, it estimates the remaining combinations which enhance subtle structures of the image. The classification is done by the *k*-means cluster analysis to identify the skin lesion on an image.

Papazoglou *et al*.^17^ proposed an algorithm for wound segmentation which requires manual input, uses the combination of RGB and CIELab colour spaces, as well as the combination of threshold and pixel-based colour comparing segmentation methods. Cavalcanti *et al*.^18^ used the independent component analysis^19,20^ (ICA) to locate skin lesions in an image and to separate it from the healthy skin. Given the ICA results, an initial lesion localization is obtained, the lesion boundary is then determined by using the level-set method with post-processing steps. Wantanajittikul *et al*.^21^ utilized the Cr values of the YCbCr colour space to identify the skin from the background in the first step, secondly the u* and v* chromatic sets of the CIELuv colour space were used to capture the burnt region, and finally, the FCM was used to separate the burn wound region from the healthy skin. Loizou *et al*.^22^ applied the snake algorithm^23^ for image segmentation to extract texture and geometrical features for the evaluation of wound healing process.

## Materials and Proposed Method

### Ethics

This study was approved by the Regional Ethics Committee in Linköping, Sweden (DNr 2012/31/31), and conducted in compliance with the “Ethical principles for medical research involving human subjects” of the Helsinki Declaration. Guardians for research subjects for this study, which was undertaken in children, were provided a consent form describing this study and providing sufficient information for subjects to make an informed decision about their child’s participation in this study. The consent form was approved by the Regional Ethics Committee in Linköping for the study. Before a subject underwent any study procedure, an informed consent discussion was conducted and written informed consent was obtained from the legal guardians attending at the visit.

### Data acquisition

All RGB images of burn patients were acquired at the Burn Centre of the Linköping University Hospital, Linköping, Sweden. The images were taken in the JPEG format utilizing the smart-phone Oneplus2 camera: 13 Mega-pixel, 6 lenses to avoid distortion and colour aberration, OIS, Laser Focus, Dual-LED flash and f/2.0 aperture. The camera was located approximately 30–50 cm from the burn wound without using the flash. Moreover, the patients were laid in a bed covered by a green sheet.

### Colour model

The green and blue components are represented by a* and b* CIELab negative values, whereas the skin and the burn wound are represented by positive values. The purpose of a colour model is to facilitate the specification of colours in some standard, generally accepted way. In essence, a colour model is a specification of a coordinate system and a subspace within that system, where each colour is represented by a single point^24^. There exist several colour models for different functions: (*i*) RGB model, (*ii*) CMY and CMYK models, (*iii*) HSI model that decouples the intensity component from the colour-carrying information (hue and saturation)^24^, (*iv*) YCbCr, CIELab, CIELuv and CIELch, where their components represent the image luminance and chromatic scales separately.

The CIELab colour model is the most complete one specified by the International Commission on illumination. The CIELab extracts the luminance and the chromatic information of an image utilizing three coordinates: the L* coordinate (L* = 0 encloses black and L* = 100 encloses white) that describes the luminance, the a* and b* coordinates that represent the pure colours from green to red (a* = −127 encloses green, a* = +128 yields red) and from blue to yellow (b* = −127 encloses blue, b* = +128 encloses yellow), respectively. Another important characteristic of this model is its uniformity, where the distance between two different colours corresponds to the Euclidean one and it coincides to the perceptual difference detected by the human visual system^25–27^. For all these reasons, the CIELab colour model was therefore chosen in this study.

Furthermore, the L*, a* and b* images were filtered with Gaussian filters in the frequency domain using the Gaussian low-pass filter function^24^. The best filters’ cut-off frequency, *σ*_0_, which keeps the 99% of the power spectrum of the zero-padded discrete Fourier transforms of the CIELab coordinates was estimated with the bisection method^28^.

### Tensor decomposition

A tensor is a multidimensional array. More formally, an *N*-way or *N*th-order tensor is an element of the tensor product of *N* vector spaces, each of which has its own coordinate system^29–31^. There are two main techniques for tensor decomposition: the CANDECOMP/PARAFAC, and the Tucker tensor decomposition. The CANDECOMP/PARAFAC, or shortly CP, decomposition factorizes a tensor into the sum of rank-one components. For example, a third-order tensor $\underline{{\bf{X}}}\in {{\mathbb{R}}}^{I\times J\times K}$ is decomposed as:

1 $$\underline{{\bf{X}}}=\sum _{r=1}^{R}\,{{\bf{u}}}_{{\bf{1}}r}\circ {{\bf{u}}}_{{\bf{2}}r}\circ {{\bf{u}}}_{{\bf{3}}r}+\underline{{\bf{E}}},$$

or element-wise:

2 $$\begin{array}{rcl}x(i,j,k) & = & \sum _{r=1}^{R}\,{u}_{1}(i,r)\,{u}_{2}(j,r)\,{u}_{3}(k,r)+e(i,j,k),\\ i & = & 1,\ldots ,I,\\ j & = & 1,\ldots ,J,\\ k & = & 1,\ldots ,K,\end{array}$$

where $\underline{{\bf{E}}}$ and $e(i,j,k)$ represent the noise or the error, $R\in {{\mathbb{N}}}^{\ast }$ represents the number of rank-one components which decompose the tensor $\underline{{\bf{X}}}$, ${{\bf{u}}}_{{\bf{1}}r}\in {{\mathbb{R}}}^{I}$, ${{\bf{u}}}_{{\bf{2}}r}\in {{\mathbb{R}}}^{J}$ and ${{\bf{u}}}_{{\bf{3}}r}\in {{\mathbb{R}}}^{K}$ for $r=1,\ldots ,R$ are the rank-one vectors which compose the factor matrices ${{\bf{U}}}_{1}\in {{\mathbb{R}}}^{I\times R}$, ${{\bf{U}}}_{2}\in {{\mathbb{R}}}^{J\times R}$ and ${{\bf{U}}}_{3}\in {{\mathbb{R}}}^{K\times R}$ respectively, and the symbol “$\circ$” indicates the vector outer product.

It is often useful to assume that the factor matrices columns are normalized to length one with the weight absorbed into a vector $\lambda \in {{\mathbb{R}}}^{R}$:

3 $$\begin{array}{rcl}\underline{{\bf{X}}} & \approx  & \sum _{r=1}^{R}\,\lambda (r)\,{{\bf{u}}}_{{\bf{1}}r}\circ {{\bf{u}}}_{{\bf{2}}r}\circ {{\bf{u}}}_{{\bf{3}}r}\\  & = & \sum _{r=1}^{R}\,\lambda (r){u}_{1}(i,r){u}_{2}(j,r){u}_{3}(k,r)\\  & = & {\rm{\Lambda }}{\times }_{1}{{\bf{U}}}_{1}{\times }_{2}{{\bf{U}}}_{2}{\times }_{3}{{\bf{U}}}_{3},\end{array}$$

where ${\rm{\Lambda }}={\rm{diag}}(\lambda )$, the symbol “×_*n*_” indicates the *n*-mode product between the core tensor and the factor matrices.

On the other hand Tucker decomposition decomposes a tensor $\underline{{\bf{X}}}$ into a core tensor $\underline{{\bf{G}}}$ multiplied (or transformed) by a matrix along each mode. For example, the third-order tensor $\underline{{\bf{X}}}\in {{\mathbb{R}}}^{I\times J\times K}$ is decomposed as:

4 $$\underline{{\bf{X}}}\approx \underline{{\bf{G}}}{\times }_{1}{{\bf{U}}}_{1}{\times }_{2}{{\bf{U}}}_{2}{\times }_{3}{{\bf{U}}}_{3},$$

where $\underline{{\bf{G}}}\in {{\mathbb{R}}}^{P\times Q\times R}$ is the core tensor, whereas ${{\bf{U}}}_{1}\in {{\mathbb{R}}}^{I\times P}$, ${{\bf{U}}}_{2}\in {{\mathbb{R}}}^{J\times Q}$ and ${{\bf{U}}}_{3}\in {{\mathbb{R}}}^{K\times R}$ are the factor matrices considered as the principal components in each mode. Equation (4) can be element-wise written as:

$$x(i,j,k)\approx g(p,q,r){u}_{1}(i,p){u}_{2}(j,q){u}_{3}(k,r)$$

where $$i=1,\ldots ,I$$, $$j=1,\ldots ,J$$, $$k=1,\ldots ,K$$, $$p=1,\ldots ,P$$, $$q=1,\ldots ,Q$$ and $$r=1,\ldots ,R$$.

**Figure 1:**
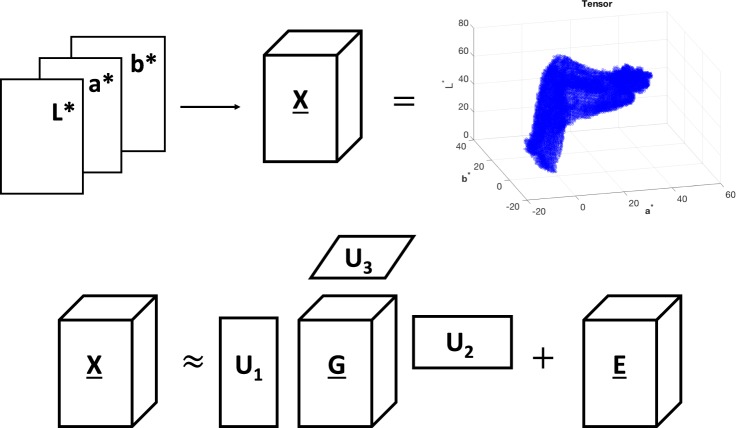
Graphical representation of the third-order CIELab tensor $\underline{{\bf{X}}}$ and its Tucker decomposition.

Both decompositions are a form of higher-order principal component analysis^30,31^. Equation (3) represents the decomposition as a multi-linear product of a core tensor and factor matrices and it is often used in signal processing. Equation (4) builds the core tensor with a different dimension for each mode and it is often used for data compression^31^. For the purpose of data compression in this study, the Tucker decomposition, known as the Tucker3 model^30,31^, is preferred to the CP. An RGB image ${\bf{I}}\in {{\mathbb{R}}}^{M\times N\times 3}$ can be converted into the CIELab space, and can be expressed as tensor $\underline{{\bf{X}}}\in {{\mathbb{R}}}^{I\times J\times K}$, where *I*, *J* and *K* are the number of grey-levels in the L* a* and b* image, respectively. Figure 1 graphically shows the Tucker decomposition of the CIELab tensor.

5 $$\begin{array}{rcl}\underline{{\bf{X}}} & = & \underline{{\bf{Y}}}+\underline{{\bf{E}}}\end{array}$$

6 $$\begin{array}{rcl} & \approx  & \underline{{\bf{G}}}{\times }_{1}{{\bf{U}}}_{1}{\times }_{2}{{\bf{U}}}_{2}{\times }_{3}{{\bf{U}}}_{3}\end{array}$$

Equations (5) and (6) can be element-wise written as:

7 $$\begin{array}{rcl}x(i,j,k) & = & y(i,j,k)+e(i,j,k)\end{array}$$

8 $$\begin{array}{rcl} & \approx  & g(p,q,r)\cdot {u}_{1}(i,p)\cdot {u}_{2}(j,q)\cdot {u}_{3}(k,r)\end{array}$$

where $\underline{{\bf{X}}}$ is the CIELab colour tensor, $\underline{{\bf{Y}}}$ is the tensor to estimate, $\underline{{\bf{E}}}$ is the tensor error, **U**_1_, **U**_2_ and **U**_3_ are the factor matrices calculated from the a*, b* and L* mode of the $\underline{{\bf{X}}}$ tensor respectively, whereas *i*, *j* and *k* are the $\underline{{\bf{X}}}$ coordinates and *p*, *q* and *r* are the core tensor $\underline{{\bf{G}}}$ ones.

Setting $\rho (i,j,k)$ as the CIELab vector module with coordinate $(i,j,k)$, two tensors, $\underline{{{\bf{X}}}_{d}}$ and $\underline{{{\bf{X}}}_{s}}$, are built in order to mix the image luminance and colour as follows:

9 $${x}_{d}(i,j,k)=\{\begin{array}{ll}0, & {\rm{if}}\,{a}^{\ast }(i) < 0\,{\rm{or}}\,{b}^{\ast }(j) < 0\,{\rm{or}}\,{L}^{\ast }(k)\le \delta \\ \tfrac{{L}^{\ast }(k)}{{\rho }^{2}(i,j,k)}[{a}^{\ast }(i)-{b}^{\ast }(j)], & {\rm{otherwise}}\end{array}$$

10 $${x}_{s}(i,j,k)=\{\begin{array}{ll}0, & {\rm{if}}\,{a}^{\ast }(i) < 0\,{\rm{or}}\,{b}^{\ast }(j) < 0\,{\rm{or}}\,{L}^{\ast }(k)\le \delta \\ \tfrac{{L}^{\ast }(k)}{{\rho }^{2}(i,j,k)}[{a}^{\ast }(i)+{b}^{\ast }(j)], & {\rm{otherwise}}\end{array}$$

where $i\in {a}^{\ast }$, $j\in {b}^{\ast }$, and $k\in {L}^{\ast }$. The tensors $\underline{{{\bf{X}}}_{d}}$ and $\underline{{{\bf{X}}}_{s}}$ in Equations (9) and (10), contain the normalized values of the addition and difference of the colour sets in proportion to the luminance, respectively. The values set to 0 indicate the background: a* and b* negative values corresponds to the green and blue components respectively; whereas ${L}^{\ast }\le \delta$ defines the dark regions where *δ* is a parameter arbitrarily chosen, in this study $\delta =10$.

Finally, the estimated $\underline{{{\bf{Y}}}_{d}}$ and $\underline{{{\bf{Y}}}_{s}}$ are re-transformed into images **Y**_*d*_ and **Y**_*s*_, the chromaticity sources of the **I** image, where the texture features are extracted.

### Tensor rank

Let $\underline{{\bf{X}}}$ be an *N*th-order tensor of size ${I}_{1}\times {I}_{2}\times \cdots \times {I}_{N}$. Then the *n*-rank of the tensor $\underline{{\bf{X}}}$, rank_*n*_$(\underline{{\bf{X}}})$, is the dimension of the vector space spanned by the mode-*n* fibres. Bro *et al*.^29,32^ developed a technique, called core consistency diagnostics (CORCONDIA), for estimating an optimal number *R* of rank-one tensor, which produces the factor matrices for the CP decomposition. Unfortunately, there is not such a single algorithm for the Tucker decomposition, so the core tensor dimensions have to be decided with a reasonable choice.

In this study, the three-mode tensor $\underline{{\bf{X}}}\in {{\mathbb{R}}}^{I\times J\times K}$ is the CIELab colour space. So, its the upper limit rank is rank$(\underline{{\bf{X}}})=[256,256,101]$, when all the luminance and chroma values define the image; whereas its lower limit is rank$(\underline{{\bf{X}}})=[1,1,1]$, when just one luminance and chroma grey-level defines the image. An intuitive choice for the rank of the CIELab tensor is the amount of grey levels of each CIELab component that defines the image **I**:

11 $$\begin{array}{rcl}{\rm{rank}}(\underline{{\bf{X}}}) & = & [P,Q,R]\\  & = & [\,{\rm{\max }}\,\{{a}^{\ast }\}-\,{\rm{\min }}\,\{{a}^{\ast }\}+1,\,{\rm{\max }}\,\{{b}^{\ast }\}\\  &  & -\,{\rm{\min }}\,\{{b}^{\ast }\}+1,\,{\rm{\max }}\,\{{L}^{\ast }\}-\,{\rm{\min }}\,\{{L}^{\ast }\}+1]\end{array}$$

As being expressed in Equations (9) and (10), the background values are set to 0, there is a further reduction which does not involve the background grey-levels of the a* and b* colour sets and the very dark regions by the L* set.

### Feature extraction

In this study, the grey-level co-occurrence matrix (GLCM)^9^ were applied on two sources of the decomposed luminance and colour components to extract the contrast, homogeneity, correlation and energy. They were calculated with a mask 5 × 5 with offsets: 0, 45, 90 and 135. The means of luminance-colour images were also included. The total of 10 values of the 5 features were extracted, 5 for each luminance-colour source. These feature vectors were used for cluster analysis to identify burn regions.

### Cluster analysis

The cluster analysis was carried out using the FCM algorithm. Since the number clusters of colours and their shades with the different luminance levels are unknown, a high value of clusters = 20 was selected for the FCM analysis, and then a cluster merging process was performed to distinguish burn from the healthy skin background regions.

**Figure 2:**
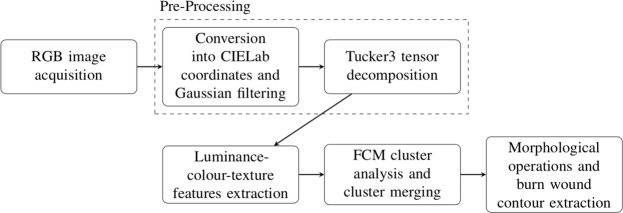
Flowchart of the proposed colour-texture segmentation of burn wounds using tensor decomposition.

Figure 2 illustrates the steps of the proposed segmentation method that works as follows. Given an RGB image of burn as the input, it is converted into CIELab space, filtered with a Gaussian filter, and two image components are computed by the Tucker3 decomposition for colour texture feature extraction. These features are the inputs of the fuzzy *c*-mean algorithm that divides the image into 20 clusters. The clusters are then merged in order to obtain three main regions of interest: burn wound, skin, and background. Once these 3 regions are obtained, the closing morphological operation is applied to obtain the burn-wound contour. However, the user has the possibility of choosing the hole filling after the closing operation.

## Results and Discussion

**Figure 3:**
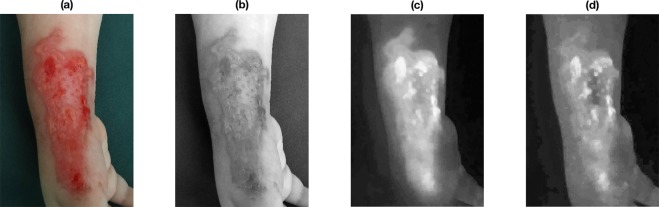
Burn wound colour image (**a**) and its CIELab coordinates: L* (**b**), a* (**c**) and b* (**d**) respectively.

**Figure 4:**
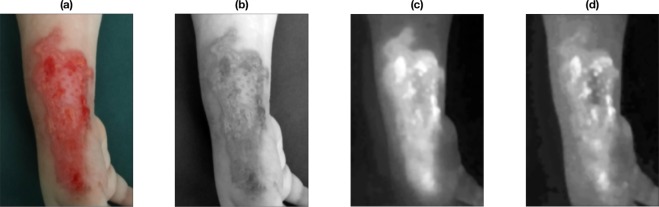
Burn wound colour image (**a**) and its CIELab coordinates (**b**–**d**) after Gaussian filtering in the frequency domain.

**Figure 5:**
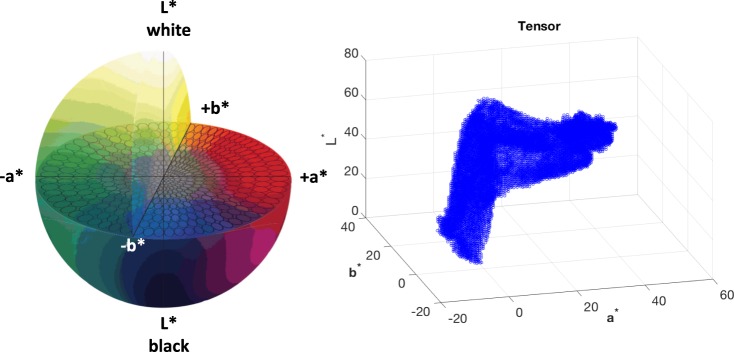
CIELab colour space and the CIELab tensor $\underline{{\bf{X}}}$ for the image in Fig. 4(a).

Figure 3 shows a burn image of $1330\times 1925\times 3$ pixels of a paediatric patient with a burn wound located on the right hand assessed 96 hours after the burn injury. The acquired RGB image was converted into the CIELab colour space with standard D65 illuminant and its components were filtered in the frequency domain with Gaussian filters, keeping the the 99% of the power spectrum of the zero-padded discrete Fourier transforms of them (see Fig. 4). Figures 3 and 4 show the effect of Gaussian filtering on the reduction of the reflection and producing a homogeneous background. This study does not consider the effect of illumination, which will be an issue for future investigation. Moreover, Fig. 5 shows the CIELab colour space and the CIELab tensor $\underline{{\bf{X}}}$ for the image in Fig. 4(a).

**Figure 6:**
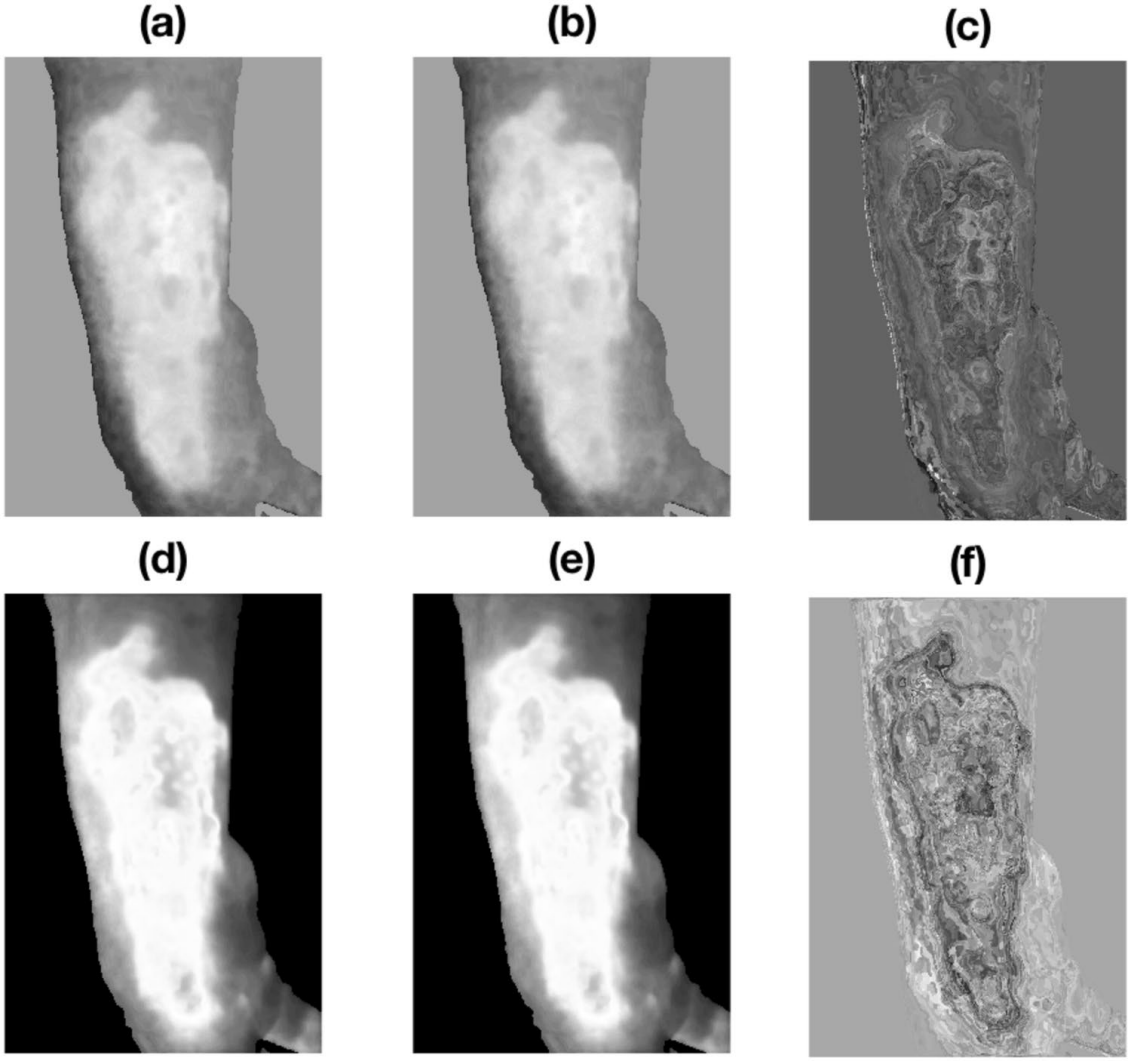
Re-transformation of images of $1925\times 1330$ images of the tensors $\underline{{{\bf{X}}}_{d}}$ (**a**) and $\underline{{{\bf{X}}}_{s}}$ (**d**), their estimations $\underline{{{\bf{Y}}}_{d}}$ (**b**), $\underline{{{\bf{Y}}}_{s}}$ (**e**) and the respective errors $\underline{{{\bf{E}}}_{d}}$ (**c**), $\underline{{{\bf{E}}}_{s}}$ (**f**) after Tucker3 tensor decompositions using rank$(\underline{{\bf{X}}})=[51,38,68]$.

Tensors $\underline{{{\bf{X}}}_{d}}$ and $\underline{{{\bf{X}}}_{s}}$ were constructed as explained in Equations (9) and (10). The tensor estimations of $\underline{{{\bf{Y}}}_{d}}$ and $\underline{{{\bf{Y}}}_{s}}$ were obtained by the Tucker3 tensor decomposition technique. The tensor rank is the amount of a*, b* and L* grey-levels: $[66,43,76]$. Since there is a background (the green blanket) and some dark areas (left side) in the image, the core tensors’ rank is reduced by using Equations (9) and (10) to rank$(\underline{{\bf{X}}})=[51,38,68]$. It should be pointed out that such decomposition was carried out on the number of unique combination of luminance and chromatic values instead on the number of pixels in the original image. In this case, the decomposition was performed on $51\times 38\times 68=131,784$ instead of $1925\times 1330\times 3=7,680,750$ pixels, resulting in a data reduction about 98.3% without losing the image information. Finally, the $\underline{{{\bf{Y}}}_{d}}$ and $\underline{{{\bf{Y}}}_{s}}$ values were re-transformed into images **Y**_*d*_ and **Y**_*s*_ with the same size of the original (see Fig. 6).

Figure 6 illustrates that the tensor decompositions can enhance the contrast of tensors $\underline{{{\bf{X}}}_{d}}$ and $\underline{{{\bf{X}}}_{s}}$ with the estimated $\underline{{{\bf{Y}}}_{d}}$ and $\underline{{{\bf{Y}}}_{s}}$ after the error eliminations $\underline{{{\bf{E}}}_{d}}$ and $\underline{{{\bf{E}}}_{s}}$, respectively. In the end, 4 and 6 show how Gaussian filtering and tensor decomposition can remove errors and/or artefacts from the image. Based on these estimated tensors, one statistical (mean) and four GLCM-based texture (contrast, homogeneity, correlation, and energy) features were extracted. These re-transformed images produce a data reduction about 25 times, from $[1925\times 1330\times 3]$ to $[385\times 266\times 3]$.

**Figure 7:**
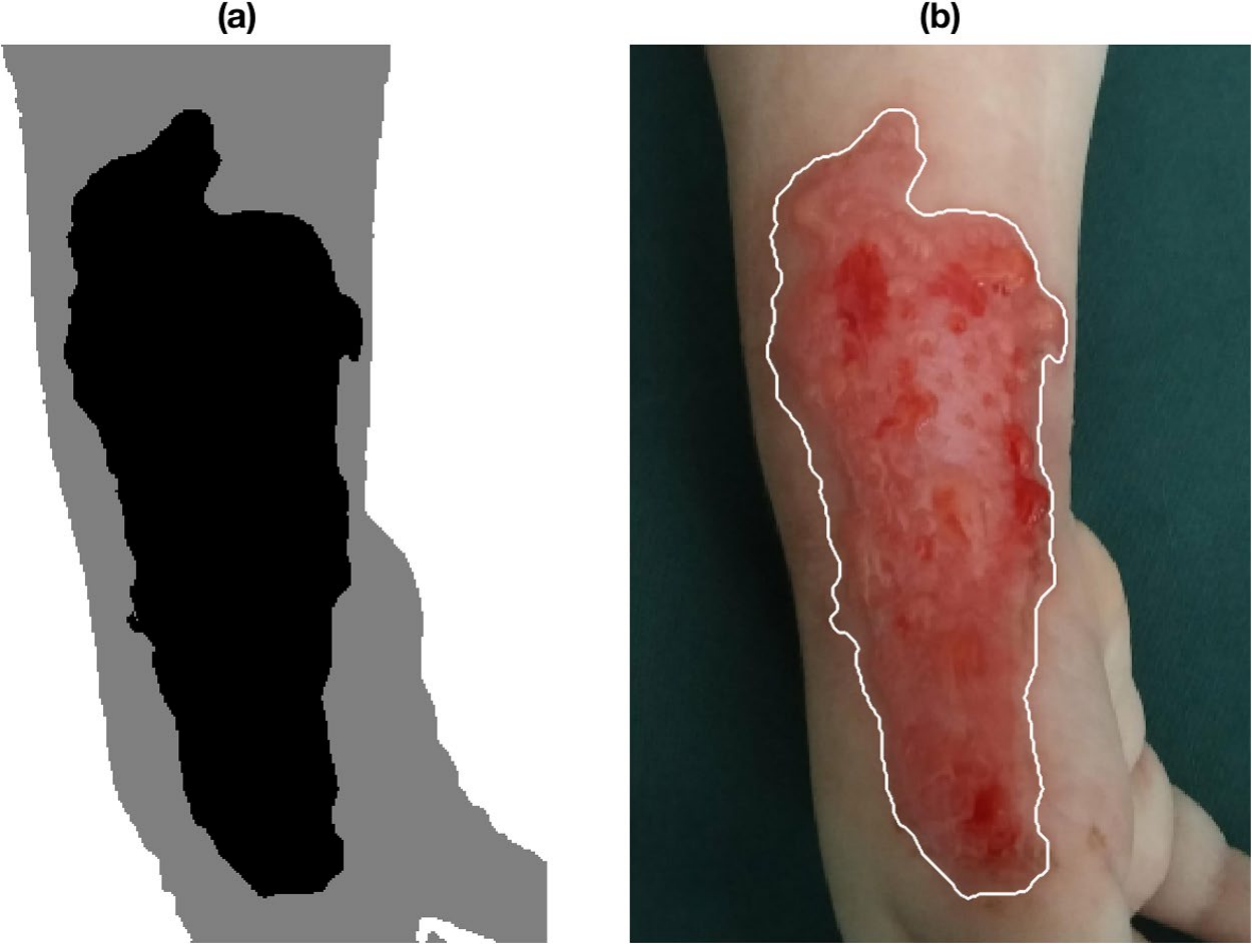
Result of merging of FCM clusters with the burn wound marked in black, the normal skin in grey and the background in white (**a**), and final segmentation result with the burn contour superimposed over the original image (**b**).

These features were used in the FCM analysis, which initially grouped the data in 20 different clusters and successively manually merged into 3 cluster: burn wound, healthy skin and background (see Fig. 7(a)). On the other hand, Fig. 7(b) shows the final image segmentation result with the burn contour superimposed over the original image. 7(a) and 7(a) are the segmentation result after a closing morphological operation with structure element disk with radius 2. Moreover it is user choice to fill or not eventual holes with another morphological operation.

**Figure 8:**
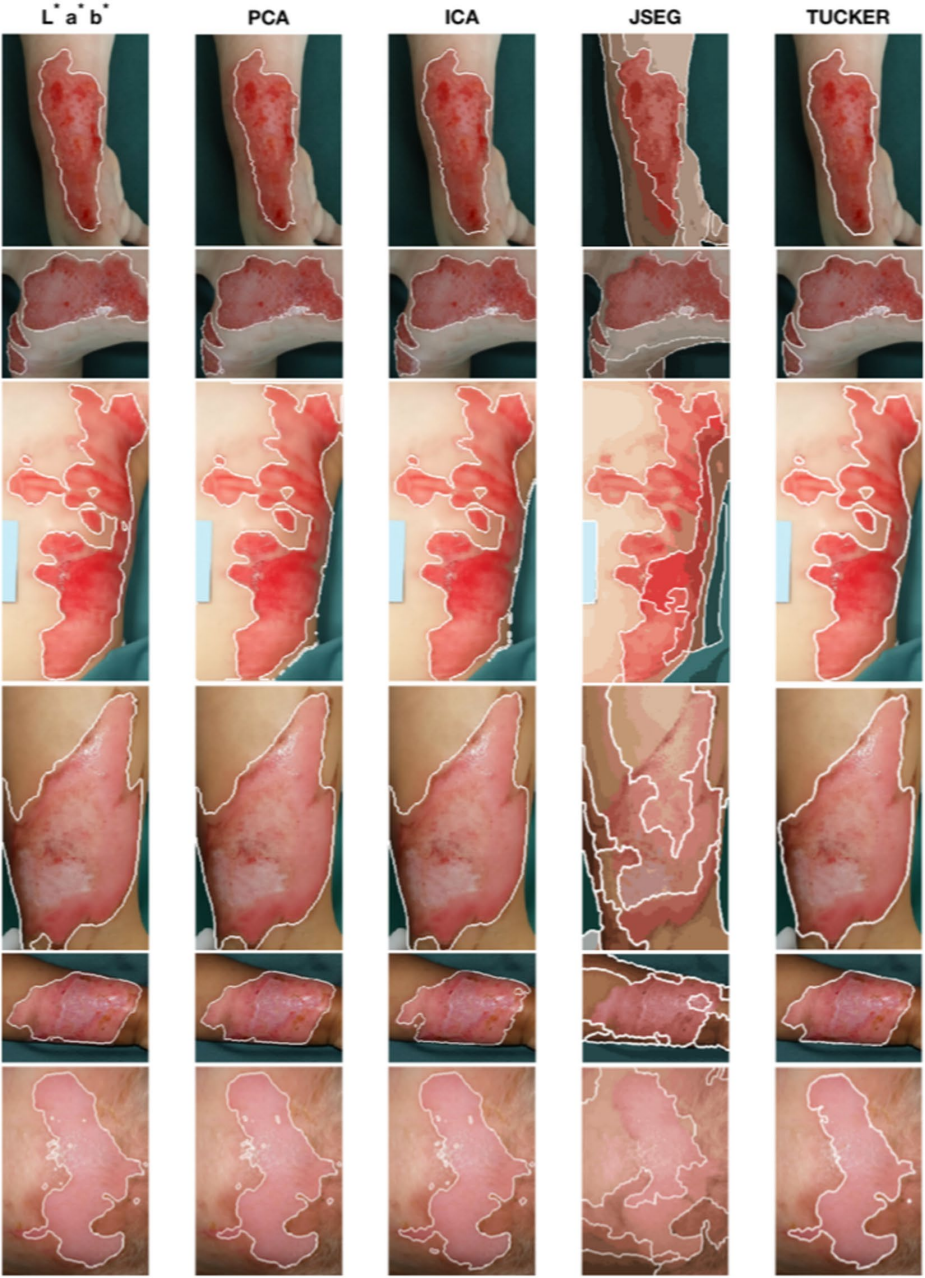
Segmentation results for six different burn wound images: the 1^st^ column shows the segmentation results using the CIELab coordinates as input of the FCM algorithm with 20 clusters; the 2^nd^ and 3^rd^ column show the segmentation result using the PCA and ICA sources as input of the FCM algorithm with 20 clusters respectively; the 4^th^ column shows the segmentation results using the JSEG technique by Deng and Manjunath; in the end, the 5^th^ column shows the segmentation results using the proposed method.

In order to compare the proposed method with others, image segmentation results were obtained using four other techniques: Gaussian pre-filtering, PCA, ICA^20^ and the JSEG^11^. Figure 8 shows six segmentation results in six rows obtained from the proposed and other four methods, which are discussed as follows. It is obvious in all cases that the JSEG can only distinguish the human body from the background but not the burn wound from the healthy skin; and therefore not further included in the following comparisons.

For the results shown in the first row, the original image is the one discussed previously with size $1925\times 1330\times 3$. The CIELab and PCA methods present under-segmented areas along the burn wound on the left side, and they took 375 and 1527 seconds for the segmentation, respectively. The ICA result is comparative with the proposed method but it required 2286 seconds for the segmentation. The proposed method successfully detects the burn wound contour after 297 seconds and using as Tucker tensor core rank: $[51\times 38\times 68]$.

Results in the second row involves a $1610\times 1835\times 3$ image, which shows three burn wounds after 96 hours of injury, located on the right side of the chest and in the right shoulder of a patient. The CIELab segmentation presents an over-segmentation along the upper side of the chest wound and required 493 seconds for the segmentation task. The PCA and ICA segmentation show over-segmented results along the right side of the wound in the bottom and took 1178 and 1705 seconds, respectively. The proposed method excludes the central white spot, caused by a specular reflection, from the segmentation and correctly identifies the burn wound contours in 358 seconds with Tucker tensor core rank: $[45\times 32\times 72]$.

The third row shows segmentation results for a $1895\times 930\times 3$ image of a burn wound after 17 hours of injury, located on the left side of the chest and lower left flank of a patient. CIELab segmentation resulted in over-segmentation as it joins a smaller burnt area separated by uninjured skin with the rest of the burn. At the same time, the CIELab segmentation also resulted in under-segmentation as it excluded a bit of the burnt areas on the left flank. The time taken for the CIELab is 239 seconds. The PCA and ICA present over-segmented results along the right side, and required 1952 and 2348 seconds for the task, respectively. The proposed method detects the burn wound contour well with a minor under-segmentation on the upper-right side of the injury in 214 seconds with Tucker tensor core rank: $[68\times 41\times 80]$.

Segmentation results shown in the forth row for an $1505\times 835\times 3$ burn image after 96 hours after injury, located next to the right ankle of a patient. Results obtained from the CIELab, PCA and ICA are similar, with over-segmentation along the left side of the image including some normal skin. These methods took 208, 800, and 3275 seconds, respectively. The proposed method detects the burn wound contour well in 118 seconds with Tucker tensor core rank: $[42\times 32\times 82]$.

The fifth row shows the segmentation results for a $965\times 1300\times 3$ image of a burn wound after 312 hours of injury, located on the left forearm of a patient. The CIELab and PCA results show some minor over-segmentation on the left side, requiring 223 and 1430 seconds for the segmentation, respectively. The ICA extracted the burn area well, but it required 3653 seconds for the task. The segmentation obtained from the proposed method is similar to the CIELab and PCA methods, but only took 115 seconds for the task and Tucker tensor core rank: $[45\times 40\times 88]$.

The sixth row presents results for a $1625\times 1140\times 3$ burn image after 24 hours of injury, located on the forehead of a patient. The CIELab, PCA and ICA segmentations show noisy results caused by the reflected light and they required 308, 958 and 2824 seconds for the segmentation, respectively. Moreover, the CIELab and PCA results are of under-segmentation on the bottom-left side of the wound. The proposed method can eliminate such noise and detects the burn wound contour well in only 185 seconds with Tucker tensor core rank: $[32\times 31\times 76]$.

Table 1 illustrates the computational times for the image segmentation obtained from different methods illustrated in Fig. 8, except for the JSEG method which produces unsatisfactory results for every images. The experimental results suggest that the proposed method can provide the best results not only in terms of segmentation accuracy but also the computational speed is approximately 10 times faster than the ICA, 5 times faster than the PCA, and 1.5 times faster than the CIELab.

**Table 1:** Computational times (seconds) for image segmentation results obtained from different methods as shown in Fig. 8, where R1, …, R6 stand for images shown in rows 1, …, row 6 of Fig. 8, respectively.

Method	R1	R2	R3	R4	R5	R6	Average
CIELab	375	493	239	208	223	308	307.66
PCA	1527	1178	1952	800	1430	958	1307.5
ICA	2286	1705	2348	3275	3653	2824	2073
Proposed method	297	358	214	118	115	185	214.5

Table 2 shows the quantitative measurements that consist of positive predicted value (PPV) and sensitivity (SEN) for a segmented image. The PPV and SEN are defined as^21^

12 $$PPV=\tfrac{{\rm{The}}\,{\rm{number}}\,{\rm{of}}\,{\rm{pixels}}\,{\rm{correctly}}\,{\rm{segmented}}\,{\rm{by}}\,{\rm{the}}\,{\rm{algorithm}}}{{\rm{The}}\,{\rm{total}}\,{\rm{number}}\,{\rm{of}}\,{\rm{pixels}}\,{\rm{segmented}}\,{\rm{by}}\,{\rm{the}}\,{\rm{algorithm}}}$$

13 $$SEN=\tfrac{{\rm{The}}\,{\rm{number}}\,{\rm{of}}\,{\rm{pixels}}\,{\rm{correctly}}\,{\rm{segmented}}\,{\rm{by}}\,{\rm{the}}\,{\rm{algorithm}}}{{\rm{The}}\,{\rm{total}}\,{\rm{number}}\,{\rm{of}}\,{\rm{pixels}}\,{\rm{in}}\,{\rm{the}}\,{\rm{segmented}}\,{\rm{region}}\,{\rm{according}}\,{\rm{to}}\,{\rm{the}}\,{\rm{expert}}}$$

**Table 2:** Quantitative measurements for image segmentation results obtained from different methods as shown in Fig. 8, where R1, …, R6 stand for images shown in rows 1, …, row 6 of Fig. 8, respectively.

	CIELab		PCA		ICA		Tucker	
	PPV	SEN	PPV	SEN	PPV	SEN	PPV	SEN
R1	0.9366	0.9449	0.9371	0.9324	0.8848	0.9952	0.9508	0.9467
R2	0.8567	0.9767	0.9334	0.9711	0.9156	0.9853	0.9444	0.9674
R3	0.9462	0.9283	0.9018	0.9688	0.8568	0.9869	0.9478	0.9562
R4	0.8474	0.9992	0.8639	0.9967	0.8581	0.9969	0.9047	0.9953
R5	0.9666	0.9825	0.9597	0.9949	0.9936	0.9004	0.9935	0.9147
R6	0.9080	0.9795	0.9149	0.9787	0.9023	0.9916	0.9518	0.9732
Average	0.9102	0.9685	0.9184	0.9737	0.9018	0.9760	0.9488	0.9589

For a perfect segmentation, $PPV=1$ and $S=1$. In case of under-segmentation, $PPV=1$ and $SEN < 1$, whereas in case of over-segmentation, $PPV < 1$ and $SEN=1$. Based on the results shown in Table 2, cases of under-segmentation are ICA with image R5 and Tucker with R5; and over-segmentation are CIELab with R6, PCA with R2, R3, R4, R5 and R6, ICA with R1, R2, R3, R4 and R6, and Tucker with R4.

Both results shown in Fig. 8 and Table 2 suggest that the proposed method provides better segmentation results for the images in the 1^st^, 2^nd^, 3^rd^ and 6^th^ row of Fig. 8. The average PPV and SEN values of the segmentations obtained from the proposed method are better than the other three methods in terms of the balance between over-segmentation and under-segmentation.

Furthermore, the proposed method is also compared with the simple linear iterative clustering (SLIC) superpixel^33^, the efficient graph-based image segmentation^34^, and the SegNet^35^ methods that are discussed as follows.

**Figure 9:**
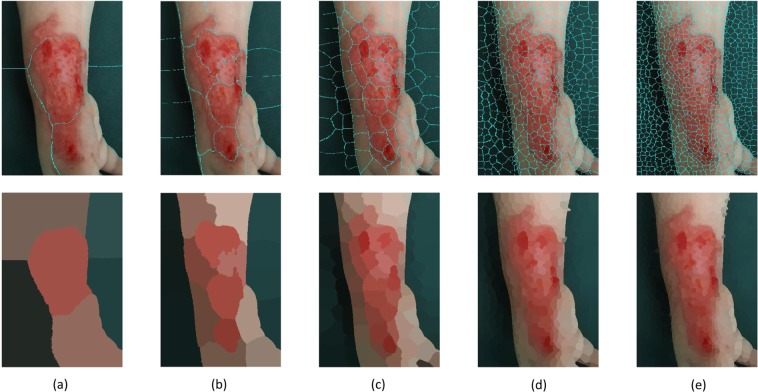
SLIC superpixel segmentation result using 5 (**a**), 20 (**b**), 100 (**c**), 500 (**d**) and 1000 (**e**) as the number of desired superpixels.

The SLIC superpixel method performs on the local clustering of CIELab values and pixel coordinates. It is fast and requires the specified number of superpixels as the input. Figure 9 shows the segmentation results of the original burn image as shown in Fig. 3(a) obtained from the superpixel method using 5, 20, 100, 500 and 1000 as the numbers of desired superpixels. It is quite obvious that the bigger the number of superpixels are, the better the segmentation result is obtained, but it is very difficult assign to which class a superpixel belongs to. The algorithm distinguishes quite well the skin from the background but then encounters a problem in classifying a superpixel as skin or burn wound, resulting in either under- or over-segmentation. Being similar to the proposed method, the SLIC superpixel technique requires a manual merging process in order to distinguish the three classes of interest. However, an advantage of the proposed method over the superpixel method is that the merging process can be carried out faster since the number of clusters specified for the proposed method is much smaller than that for the SLIC superpixel technique to achieve a good final segmentation result as shown in Fig. 7(a).

**Figure 10:**
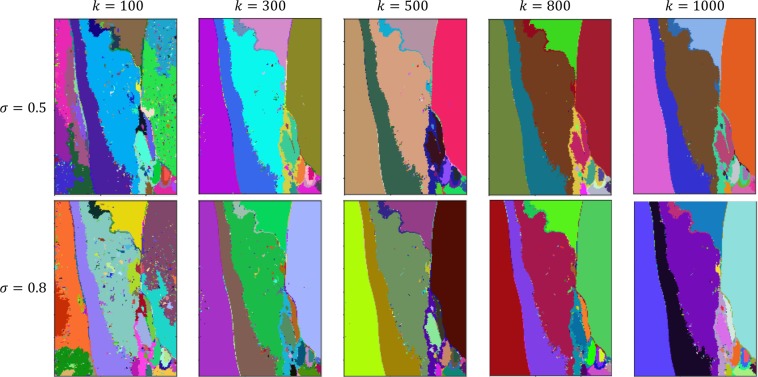
Efficient graph-based image segmentation results using various values of *σ* and *k*.

The efficient graph-based image segmentation method defines a predicate to highlight the boundary between 2 or more regions using a graph-based representation of the image of interest. Figure 10 shows the segmentation results of the original burn image as shown in Fig. 3(a) obtained from the graph-based image segmentation method, where its input parameters $\sigma =0.5,0.8$, and $k=100,300,500,800,1000$, where σ is the standard deviation of the Gaussian filter in the pre-processing and *k* is a scaling parameter. It is easy to observe that all the results are not satisfactory as they were largely over-segmented.

The SegNet consists of an encoder network and a corresponding decoder network followed by a pixel-wise classification layer. It is composed of an encoder sub-network and a corresponding decoder sub-network. The depth of such network is specified by a scalar *D* that determines the number of times an input image is downsampled or upsampled as it is processed. The encoder network downsamples the input image by a factor of 2^*D*^. The decoder network performs the opposite operation and upsamples the encoder network output by a factor of 2^*D*^. Two types of the SegNet were designed: *i)* encoder and decoder with *D* = 4, and *ii)* the network is initialized using the VGG-16 weights and *D* = 5. Using these two networks with 11 images as training and 2 as testing, the accuracies achieved after 10 epochs and with learning rate = 10^−3^ are 26% and 25%, respectively. A reason for the poor results produced by SegNet can be that SegNet was primarily designed for scene understanding applications (road and indoor scenes), while the data domain in this study is medical imaging of burn wounds. Another possible reason is the very small training sample size (11 images) used for training the SegNet in this study, which was not sufficient for the deep network to capture the feature map information for correct learning, particularly the vague boundary information between the skin and burn areas.

**Figure 11:**
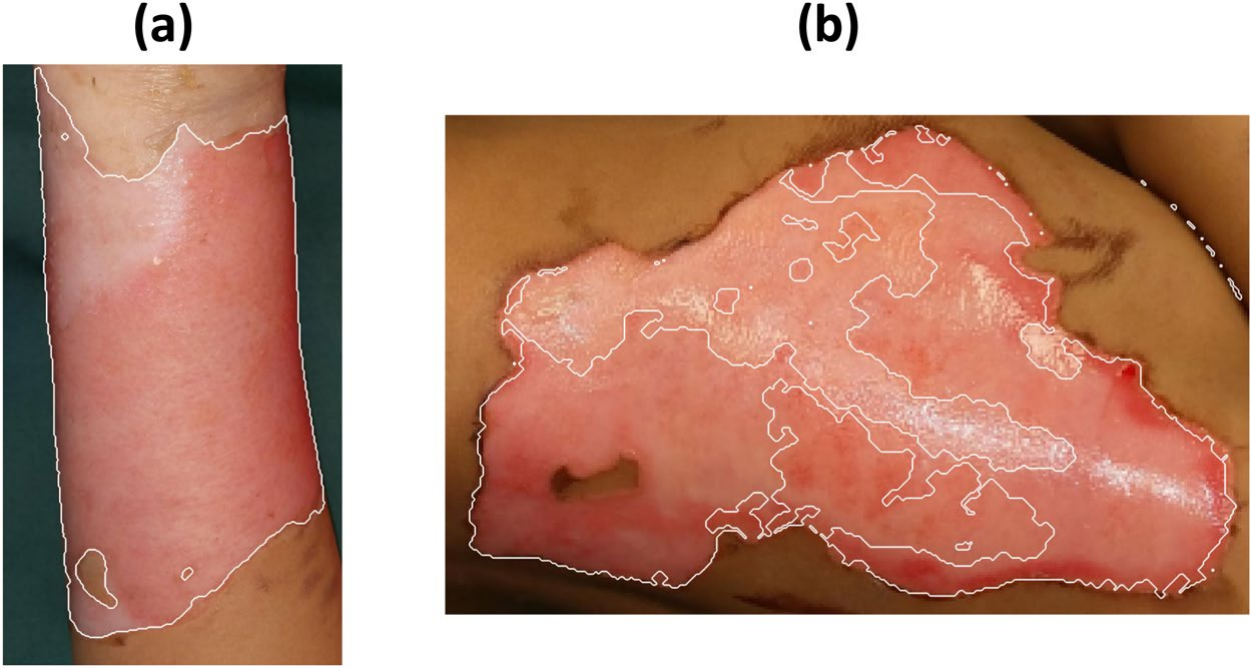
Example of desirable (**a**), and undesirable (**b**) segmentation results with automatic merging of fuzzy clusters, depending on sufficient training data.

It should be pointed out that good outcome of automated merging of the fuzzy clusters obtained from the FCM depends on sufficient training data for various types of burn, as shown in Fig. 11, where the assignment of new fuzzy clusters to the trained ones is based on the minimum distance criterion. A dataset with about 220 clusters centres (8 belong to the background, 135 to healthy skin, and 77 to burn wound) was developed and as reference to assign each new cluster centre extracted from an image under the current analysis to the class of the reference centre that has the minimum distance. As the number of the reference cluster centres are limited, the automated merging fails sometimes. Therefore, the user has the opportunity to do this process manually and then adds the new labelled cluster into the reference set.

Regarding the sufficient data and training process, at present it is difficult to optimally determine how much more data would be needed for the good training of the proposed algorithm. In fact, sample size planning for classifiers is an area of research in its own right. Most methods of sample size planning for developing classifiers require non-singularity of the sample covariance matrix of the covariates^36^. In biomedicine, methods for sample size planning for classification models were developed on the basis of learning curves that show the classification performance as the function of the training sample size to appropriately select the sample size needed to train a classifier. However, these methods require extensive previous data that attempted to differentiate the same classes^37^, or suggest sizes between 75–100 samples to achieve only reasonable accuracy in the validation^38^. This issue will certainly be investigated in our future research when more clinical data become available.

## Conclusion

The proposed method has been shown to be able to extract burn wounds from the complex background with relatively fast computational time. The tensor decomposition is independent from the camera resolution, because it works on the CIELab tensor model instead on the number of pixels of the image. The proposed method results in a big data reduction without any information lost for the image source estimation, and therefore applicable for real-time processing. The CIELab, PCA and ICA do not consistently provide good segmentation results over various burn images, showing over/under-segmentation errors. Moreover, these techniques require longer computational times than the proposed method.

Besides, the fuzzy burn wound centres extracted by the FCM during the cluster analysis, in this paper used to distinguish partial-thickness burns from normal skin and 1^st^ degree burns, but they could also be used to identify the depth of the burn and classify it into: superficial partial-thickness burn, deep partial-thickness, and full-thickness burns. The 1^st^ degree burns are not included in the total area of burn estimation and should therefore not be included in this estimation.

The strategy for colour image segmentation with fuzzy integral and mountain clustering^39^ which does not require an initial estimate of the number of fuzzy clusters, is worth investigating for improving the proposed approach in terms of limited training data for cluster merging. It would be desirable to utilize the proposed method for segmenting images captured with a polarized camera that can eliminate the light reflection problem, and try to extract features on the CIELab tensor instead of the re-constructed images. As another issue for future research, it is worth investigating the segmentation of burn areas on 3D images to include curves and depth to further improve the segmentation accuracy. Furthermore, there exist many image segmentation techniques such as semantic segmentation^35,40^, superpixels segmentation^33,41^, spectral clustering^42^, fully connected conditional random fields^43^, and mask R-CNN^44^. However, all these techniques require a huge amount of training data to achieve a high degree of accuracy. To utilize such techniques, developing a method for simulating and generating a large quantity of burn images would be an important area of research to pursue in our future work.
